# Exploring post-operative pain management practices for improved outcomes among nurses in public hospitals in West Shewa, Ethiopia: a multicenter observational study

**DOI:** 10.3389/fpain.2025.1571968

**Published:** 2026-01-30

**Authors:** Firaol Regea Gelassa, Ababe Dechasa, Desalegn Abdissa, Tesfu Zewdu, Lammi Atomsa, Abdo Kurke

**Affiliations:** 1 Department of Nursing, School of Health Sciences, Ambo University, Waliso, Ethiopia; 2 Department of Nursing, College of Health Science, Ambo University, Ambo, Ethiopia; 3 Department of Medicine, College of Medicine and Health Science, Ambo University, Ambo, Ethiopia; 4 Department of Nursing, College of Health Sciences, Salale University, Fiche, Ethiopia; 5 Department of Nursing, School of Nursing and Midwifery, Wollega University, Nekemte, Ethiopia

**Keywords:** post-operative pain management, nurses, practice, public hospitals, Ethiopia

## Abstract

**Background:**

Effective postoperative pain (POP) management is crucial to enhance satisfaction and recovery. Nurses play a significant role in providing effective pain management, making it crucial to assess their practices. However, most prior research in Ethiopia relied on self-administered tools to evaluate nurses' POP management, potentially introducing bias. This observational study addressed this gap by exploring POP management practices and associated factors among nurses in public hospitals in the West Shewa zone.

**Method and materials:**

A facility-based cross-sectional study incorporating both self-reported and observed quantitative measures was conducted among 377 randomly selected nurses in public hospitals in the West Shewa zone, Ethiopia, from June 1 to August 30, 2020. Data were collected using a standardized interviewer-administered questionnaire and an observational checklist. Non-participant observation was done three times by trained BSc degree nurses. Data quality was ensured, and analysis was done using SPSS version 26. The logistic regression analyses were done to identify factors independently associated with nurses' POP management practice. Adjusted odds ratio (AOR) with 95% CI was estimated to measure the strength of the association. The level of statistical significance was set at a p value <0.05.

**Result:**

Only 25.72% (95% CI: 23.4–32.6) of observed nurses demonstrated good postoperative pain management practices. Factors significantly associated with good practice included being female [AOR: 2.56, 95% CI: 1.78–4.79], using standardized pain assessment tools [AOR: 2.94, 95% CI: 1.65–5.86], working in hospitals with pain management policies [AOR: 3.10, 95% CI: 1.65–5.86], employment in surgical units [AOR: 2.93, 95% CI: 1.27–6.80], and having received relevant training [AOR: 2.28, 95% CI: 1.46–7.40].

**Conclusion:**

Only a quarter of nurses demonstrated good postoperative pain management practices. Female nurses, use of standardized pain assessment tools, presence of formal hospital pain management policies, work in surgical units, and prior training were key enablers. To improve practice, hospitals should provide regular in-service training focused on evidence-based postoperative pain assessment and intervention, implement standardized pain assessment tools, and enforce comprehensive institutional policies guiding pain management across all wards.

## Background

Post-operative pain (POP) is a common form of acute pain that occurs after surgery and results from tissue injury caused by incision, dissection, traction, and manipulation during the procedure (1, 2). It is defined as an unpleasant sensory and emotional experience related to actual or potential tissue damage, and it is acknowledged whenever the person experiencing it reports its presence (3, 4). Pain can further be categorized based on its etiology, duration, and intensity (5).

Pain relief has been recognized as a basic human right and is also regarded as the “fifth vital sign.” As such, it requires regular assessment and effective management. Nurses, being at the bedside around the clock and serving as the primary point of contact between patients and other health professionals, play a vital role in this process (6–8). According to the American Nurses Association (ANA), their responsibilities include comprehensive pain assessment, planning individualized interventions, administering treatment, evaluating patient responses, and making necessary adjustments to ensure adequate pain control (7).

Effective POP management is a critical component of quality surgical care, as it has a direct impact on recovery, complication rates, patient satisfaction, and overall health outcomes (8). Conversely, inadequate pain control is associated with delayed recovery, prolonged hospitalization, and diminished quality of life (9). Despite the importance of their role, evidence shows that nurses' practices in POP management are often inconsistent or inadequate (10).

Assessing nurses' post-operative pain management practices is essential. However, most previous studies have relied on self-administered questionnaires, which may be prone to bias (11, 12). To address this gap, our broader research employed a quantitative design supported by direct observation. The current manuscript reports the observational findings, which provide a more objective assessment of actual nursing practices in the management of postoperative pain. Therefore, this study aims to explore the current practices of postoperative pain management among nurses in public hospitals in West Shewa, Ethiopia, with the goal of generating evidence that can inform targeted interventions and improve patient outcomes.

## Methods and materials

### Study setting and populations

A facility-based cross-sectional study incorporating both self-reported and observed quantitative measures was conducted in public hospitals of West Shewa Zone from June 1, 2020, to August 30, 2020. Data were collected using two approaches: a structured self-administered questionnaire and direct observation of nursing practice.

The study was conducted in all eight public hospitals in West Shewa Zone, Oromia Regional State, Ethiopia. West Shewa is located approximately 114 km from Addis Ababa. These hospitals provide surgical and postoperative care and were included because post-operative pain management is routinely performed at hospital settings.

The study population included all nurses working in adult surgical wards, adult ICUs, operating rooms, orthopedic wards, recovery rooms, medical wards, emergency, and obstetrics/gynecology wards in the eight public hospitals of West Shewa Zone. Nurses with no direct patient contact, ward head nurses, and students were excluded. A proportional allocation method was used to determine the number of nurses sampled from each hospital based on the total number of eligible nurses. Within each hospital, simple random sampling (lottery method) was applied to select individual participants, ensuring each nurse had an equal chance of inclusion.

### Sample size and sampling procedures

The sample size was calculated via a single population proportion formula with the following assumptions: proportion of nurses, who had good practice (65.2%) on postoperative pain management, taken from quantitative study conducted among nurses in Arsi zone Ethiopia (36), a 5% margin of error, and 95% confidence in level. Finally, with the addition of a 10% nonresponse rate, 377 nurses in the study area were considered to have participated in the study.

First, to get adequate sample size, all hospitals were included in the study. After that, the total calculated sample size was proportionally allocated to each hospital based on the number of their nurses. Then finally, the random sampling method was used to select those proportionally allocated study participants from each hospital by lottery method.

### Data collection tool and procedures

In the current study, Data were collected using structured self-administered questionnaires and direct observation checklists. The questionnaires captured nurses' knowledge, attitudes, and self-reported practices, while the observational checklist objectively assessed actual nursing practices in postoperative pain management. To facilitate this, we developed a structured observational checklist by reviewing relevant literature (See Supplementary File 1). Additionally, self-administered questionnaire was used assess variables related to post-operative pain management (See Supplementary File 2). The consistency of the questionnaire prepared in English was translated into the local language (Amharic and Afan Oromo), and the questionnaire was back-translated to English to check its consistency.

The observational checklist and structured self-administered questionnaire were adapted from previously published studies on postoperative pain management practices (13, 14). To ensure data quality, all data collectors received standardized training on checklist use. Inter-rater reliability was evaluated during a pilot observation of 10% of the sample, with each nurse independently observed by two raters. Agreement between observers was measured using Cohen's kappa coefficient (*κ* = 0.87), indicating strong consistency. Any discrepancies were discussed, and the checklist was refined accordingly. Both the checklist and questionnaire were reviewed by a panel of nursing and pain management experts to ensure content validity. A pretest involving 10% of nurses in a hospital not included in the main study assessed clarity, feasibility, and reliability. The internal consistency of the quantitative questionnaire was confirmed with a Cronbach's alpha of 0.82.

The observational check list pharmacological and non-pharmacological methods of pain management. The observation was done three times by using validated observational checklist while each study participant was giving care for different three patients after a surgical procedure. Data were collected in working hours of two shifts (morning and afternoon) including medication time at (12:00 Am, 12:00 MD, 2:00 Pm and at 6:00 Pm). In order to minimize the *Hawthorne effect,* observers did not provide details of the study procedure for nurses. Ward nurses were assigned to six and more beds; first, we identified whom nurse assigned at which bed and on what shift from ward head nurses. Then the identified nurses were observed just starting from, when they received or admitted the patient from operation room (OR) after surgery to the bed where they assigned. In clinical observation each selected participant was observed three times on three different patients after surgery, by using observational checklist. After data collection from observation was finalized, self-administered questionnaire was distributed. Structured self-administered questionnaire was used to collect data about socio demographic information, attitudes, knowledge and practice of nurses regarding post-operative pain management.

Two trained BSc nurses for self-administered and two public health officers for observational data collection were participated. From the two observational data collectors one was an assistant and involved each time together in each and every observation to prevent the observer bias. The principal investigator and one MSc nurse was supervised the process of data collections.

The quality of the data was maintained by providing them with two days of training on techniques and approaches for data collection. A pretest was also conducted among 19 working in adjacent health facilities to ensure that the data collectors and respondents understood the questions. Accordingly, appropriate amendments were made to the questionnaire after the pretest. The data collection process was supervised, and the completed questionnaires were cross-checked for completeness and consistency. Finally, the data were edited for possible errors, double entered into Epi-Data version 3.1 to control for errors that occurred during data entry, and cleaned for missing values and outliers in SPSS version 26.

### Measurements

#### Observational practice

To determine the level of practice, we assessed distributions of knowledge and practice scores for normality using the Shapiro–Wilk test and histogram plots. The scores were approximately normally distributed, and then the score of mean value and above of the total performance were labeled as good practice whereas the score of performance below the mean value was regarded as poor practice (13).

#### Knowledge

Is measured by fifteen items in yes/no format. Correct answer was given “1” and “0” was given for incorrect and for not sure. Then a total score was computed out of fifteen marks (with the range of 0–15) those who scored mean and above the mean were labeled as have adequate knowledge where as those who scored below the mean value have inadequate knowledge about post-operative pain management (14).

### Data analysis

The collected data were edited, coded and entered into Epi-Data version 3.1 and then exported to SPSS 26 for analysis. Frequencies, proportions, and summary statistics were computed to describe the study population in tables, graphs and charts. Prior to logistic regression, data were screened for outliers and multicollinearity. Outliers were assessed using boxplots and standardized residuals; observations with standardized residuals exceeding ±3 were examined, and no cases were removed as all were plausible. Multicollinearity among independent variables was assessed using the Variance Inflation Factor (VIF) and tolerance statistics, with all VIF values <10 and tolerance >0.1, indicating no multicollinearity issues. Bi-variable and multivariable logistic regressions were then computed to determine the presence and degree of association between the independent and dependent variables. *P* < 0.25 was considered statistically significant for bi-variable analysis to select variables as candidates for multivariable logistic analysis, and a *p* value <0.05 and 95% CI were used to judge statistical significance.

## Results

### Sociodemographic characteristics of the participants

A total of 377 participants were observed, yielding a response rate of 100%. The majority, 227 (60.2%), were male. Most respondents, 240 (63.7%), were aged between 26 and 34 years. Out of the total participants, 200 (53.1%) were married. A substantial proportion, 346 (91.8%), held a bachelor's degree. Among the respondents, 234 (62.1%) had served for less than five years, and over one-fourth, 105 (27.9%), were currently working in the surgical ward. Additionally, slightly more than half, 199 (52.8%), had less than one year of experience in postoperative care (Table 1).

**Table 1:** Sociodemographic characteristics of respondents working in public hospitals in the West Shewa zone, Oromia region, Ethiopia.

Variables	Category	Frequency(n=377)	Percentage (%)
Sex	Male	227	60.2
	Female	150	39.8
Age	<25	71	18.8
	26–34	240	63.7
	>35	66	17.5
Educational level	Diploma	28	7.4
	Bachelor degree	346	91.8
	Masters	3	0.8
Years of experience	<5	234	62.1
	6–9	75	19.9
	>10	68	18
Work experience in surgical unit (in years)	<1	199	52.8
	2–4	140	37.1
	>5	38	10.1
Current area of practice	Medical ward	65	17.2
	Emergence ward	70	18.6
	Ob/Gyne ward	65	17.2
	OR and recovery	72	19.1
	Surgical ward	105	27.9

### Organizational characteristics related to postoperative pain management

Nearly three-fourths (273, 72.4%) of nurses in the hospitals had not received any training on postoperative pain management. Additionally, 221 nurses (58.3%) reported lacking access to pain management guidelines in their hospitals. Among the 156 nurses who did have access to the guidelines, 80 (51.3%) reported reading them consistently, 30 (19.2%) referred to them monthly, 19 (12.2%) quarterly, and 27 (17.3%) annually (see Table 2).

**Table 2:** Organizational factors influencing postoperative pain management practices among respondents in public hospitals, West Shewa zone, and Oromia region, Ethiopia.

Organizational related factors	Category	Frequency	Percent
Previous training on POP	Yes	104	27.6
	No	273	72.4
Means of receiving training	Lecture	59	15.6
	Course	39	10.3
	Conference	2	0.5
	Workshop	4	1.1
Access to guidelines related to POP management in hospital	Yes	156	41.4
	No	221	58.6
Frequency of Read guidelines	Always	80	21.2
	Monthly	30	8
	Quarterly	19	5
	Yearly	27	7.2

### Nurses' perceived barriers to optimal postoperative pain management

Participants identified several barriers to effective postoperative pain management. The most commonly reported barriers included the lack of a standard pain assessment tool in hospitals (71, 18.8%), lack of training (68, 18%), high nursing workload (42, 11.1%), patients' inability to report pain (14, 3.7%), and the absence of pain management guidelines (13, 3.4%).

Among nurses who reported assessing pain, only 110 (29.1%) documented their findings. Of these, 42 (11.1%) consistently documented results, while 68 (18%) did so occasionally. Among the 267 nurses who did not document findings, the main reasons reported were high nursing workload (119, 31.6%), lack of familiarity with pain assessment tools (83, 22%), and the absence of designated areas for charting (65, 17.2%) (see Table 3).

**Table 3:** Distribution of nurses’ perceived barriers to optimal postoperative pain management practices in public hospitals, West Shewa zone, Oromia region, Ethiopia (*n* = 377).

S. No.	Variables	Category	Frequency (*n*)	Percentage (%)
1	Pain assessment for patients able to communicate	Yes	327	86.7
		No	50	13.3
2	Use of pain assessment tool	Yes	169	44.9
		No	158	41.9
3	Type of pain assessment tool used	Verbal Rating Scale	114	30.2
		Numeric Rating Scale	50	13.3
		Visual Analogue Scale	5	1.3
4	Frequency of use of pain assessment tool	Always	44	11.7
		Sometimes	125	33.2
5	Perceived barriers to optimal postoperative pain management	Lack of standard pain assessment tool	71	18.8
		Lack of training on pain management	68	18.0
		Nursing workload	42	11.1
		Patient inability to communicate	14	3.7
		Lack of pain management guideline in hospital	13	3.4
6	Documentation of pain assessment	Yes	110	29.1
		No	267	70.9
7	Frequency of documentation of pain assessment	Always	42	11.1
		Sometimes	68	18.0
8	Reasons for not documenting pain assessment	Nursing workload	119	31.6
		Lack of familiarity with assessment tool	83	22.0
		No designed area for charting	65	17.2

### Post-operative pain management practice

**Figure 1:**
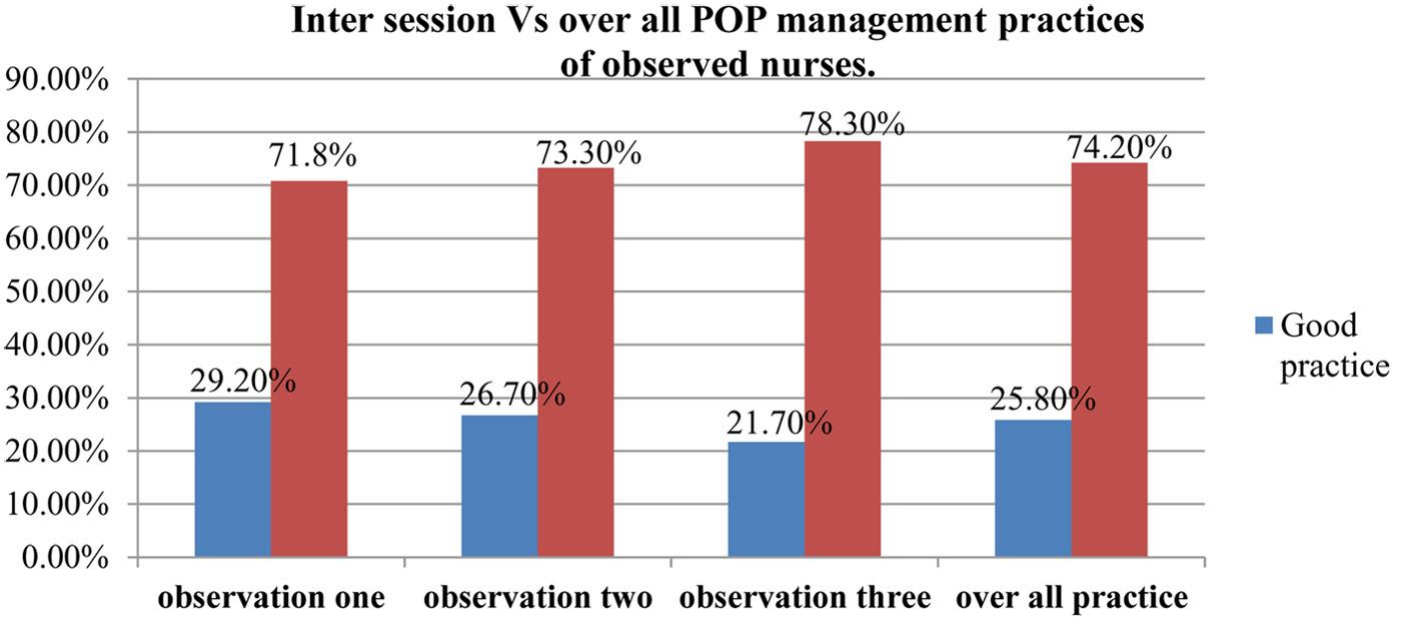
Postoperative pain management practice level of observed nurses in each observational session vs. the overall practice at four selected public hospitals in west Shewa zone, Oromia, Ethiopia 2020.

Observational data were collected from 377 nurses while they were giving care to patients after surgery for three months at four randomly selected hospitals (AURH, AGH, Gedo general Hospital and Jaldu general Hospitals). The occurrence of postoperative pain management practices were observed by using validated observational check list and three round observations were done, which constitute to 1137 clinical observations. From those observed nurses, 245(65%) and 132(35%) of them were working at Surgical and Gynecology units of the hospitals respectively. The mean score for compliance of observed practices of postoperative pain management was 3.38 with standard deviation of 1.85. Among the observed participants about, 110 (29.2%) at observation one, 101 (26.7%) at observation two and, 82(21.7%) at observation three were scored above the mean. And of the overall three sessions of the observations, only 97(25.72%) of the observations were scored above the mean value. Thus, as a result of all observations, about 25.72% [95% CI: (21.1, 30.3)] of all observed participants had good practice whereas the remaining 280(74.2%) had poor practice (see Figure 1).

To evaluate nurses' postoperative pain management practices in hospitals, an observational checklist with 11 questions covering pharmacological and non-pharmacological methods was used. The findings showed that standardized pain assessment tools were used in 106 (28.6%) of observations, with the verbal rating scale being the most common tool (88, 82.5%).

Regarding documentation, only 64 (16.9%) of those who used tools documented their findings. Timely medication administration was observed in 225 (59.7%) cases. However, only 87 (23.1%) evaluated the effectiveness of previous analgesia during subsequent administrations, and 114 (30.3%) provided pain medication before painful events like wound care or dressing.

Non-pharmacological pain-relief interventions were observed in 110 (29.2%) of cases. Among these, positioning was practiced in (45, 41%) of observations, emotional support in (34, 31.4%,) comforting in (17, 15.2%,) and supportive touch in (14, 12.4%).

### Factors associated with post-operative pain management among nurses working in public hospitals

In a multivariable logistic regression analysis, several factors were found to be significantly associated with post-operative pain management practice among nurses working in West Shewa Zone public hospitals, these factors included gender of participants, use of the standardized pain assessment tool, presence of pain management policy in the unit, working in the surgical unit, having post-operative pain management training were significantly associated with the nurses‟ postoperative pain management practice (see Table 4).

**Table 4:** Logistic regression analysis of factors associated with postoperative pain management practices among nurses in public hospitals, West Shewa zone, Oromia region, Ethiopia.

Variables name	Variables category	Practice status		COR	AOR	*P*-value
		Good	Poor			
Age	<25 years	51	20	1.0		
	26–34 years	151	89	0.67 (0.37–1.19)*	0.706 (0.34–1.47)	0.350
	>35 years	47	19	0.97 (0.46–2.04)	0.387 (0.12–1.29)	0.123
Sex	Male	97	53	1		
	Female	75	152	3.71 (1.45, 6.78)*	2.56 (1.78, 4.79)	**0**.001**
Marital status	Single	109	68	0.69 (0.45–1.054)*	1.04 (0.57–1.9)	0.90
	Married	140	60	1.0	1	
Level of education	Degree & above	234	115	1.76 (0.81–3.83)*	2.495 (0.86–7.21)	0.09
	Diploma	15	13	1	1	
Year of experience	<5	140	94	1.0	1	
	6–9	60	15	2.686 (1.4–5.1)*	2.3 (0.93–5.68)	0.071
	>10	49	19	1.732 (0.96–3.13)*	1.61 (0.499–5.22)	0.42
Working Unit	Medical ward	31	34	1.0		
	Emergence ward	42	27	1.7 (0.86–3.39)*	1.62 (0.689–3.83)	0.27
	Gyne ward	48	17	3.09 (1.48–6.47)*	2.342 (0.92–5.99)	0.08
	OR and Recovery	45	27	1.828 (0.92–3.61)*	1.68 (0.71–4.0)	0.24
	Surgical ward	8	2	3.96 (2.023–7.742)*	2.934 (1.27–6.795)**	**0**.015**
Training on POPM	Yes	78	13	4.035 (2.143–7.599)*	3.289 (1.461–7.403)**	**0**.004**
	No	171	115	1.0	1	
Presence of PM policy in unit	Yes	133	23	5.23 (3.13–8.76)*	3.11 (1.65–5.86)	**0**.001**
	No	116	105	1		
Using standard pain assessment tool	Yes	169	38	5.003 (3.15–7.95)*	2.939 (1.65–5.23)	**0**.001**
	No	80	90	1.0		

AOR, Adjusted odds ration; COR, Crude odds ratio, 1.00 = Reference.

Bold values indicate statistically significant associations at *p* < 0.05.

* *p*-value < 0.25,

** Statistically significant at *P* < 0.05.

In this study, female nurses were nearly about 2.5 times more likely to engage in effective pain management practices compared to males (AOR: 2.56; 95% CI: 2.1.78, 4.79; *P* < 0.001). additionally, nurses working in surgical unit were 2.93 times more likely to demonstrate proper practices compared to those working in other unit (AOR: 2.93; 95% CI: 1.27,6.79; *P* < 0.0015). Similarly, participants who used standardized pain assessment tools during pain management were about 3 times more likely to practice effectively compared to those who did not use such tools (AOR: 2.935; 95% CI: 1.65,5.227; *P* < 0.0001). Furthermore, nurses working in institutions with established pain treatment policies and guidelines were 3.1 times more likely to engage in good pain management practices compared to those without access to such resources (AOR: 3.1; 95% CI: 1.65, 5.86; *P* < 0.001). Additionally who had training on post-operative pain management were 2.28 (1.46, 7.4). These findings emphasize the importance of experience, specialization, standardized tools, and institutional support in enhancing pain management practices.

## Discussion

The aim of this study was to explore the level of nurses' practice toward postoperative pain management and associated factors among nurses working west Shewa Zone public hospitals. In the current study, only 25.72% (95% CI: (21.1, 30.3) of nurses demonstrated good (standard) postoperative pain management techniques. Unlike many previous studies that relied on self-reported questionnaires to assess postoperative pain management practices, our study employed structured observations to capture actual nursing behavior. For instance, studies conducted in Ethiopia and other low-resource settings using self-administered questionnaires have reported higher rates of “good” pain management practice among nurses, ranging from 45% to over 60% (11, 12).

In contrast, our observational findings indicate a lower proportion of good practice, reinforcing the concern that self-reported measures may overestimate actual clinical behavior. These results underscore the importance of observational approaches to reduce potential self-report bias and provide a more objective assessment of nursing practice.

The level of practice reported in this study was comparable with study conducted in Vietnam (27%), Ghana (28.2%), Hawassa, Southern Ethiopia (22.5%), and Wolaita Sodo referral hospital (23.7%) (12, 13, 15, 16). The similarity of the finding might be attributed to shared characteristics, such as lack of training opportunities, cultural perceptions of pain, and insufficient institutional policies. These results highlight the need for context-specific approaches to enhance pain treatment procedures worldwide.

The relatively low proportion of nurses demonstrating good postoperative pain management practice in this study may be due to multiple contextual factors. High patient loads, limited human and material resources, and staff fatigue in many public hospitals reduce the time available for comprehensive pain assessment and intervention. Restricted access to opioid medications further limits effective pharmacologic pain control. Additionally, cultural beliefs that consider pain a natural part of recovery, along with limited in-service training and insufficient institutional guidelines, contribute to suboptimal adherence to evidence-based practices. These challenges, reported in other low- and middle-income countries, highlight the need for targeted policy support, capacity-building, and improved resource allocation to enhance postoperative pain management (17–20).

The level of post-operative pain management practice observed in the current study is lower compared to studies conducted in various regions, including the United States (68%), Bharatpur, Nepal (52.7%), Rwanda (88%), Amhara regional state referral hospitals in Ethiopia (47.6%), Addis Ababa public hospitals in Ethiopia (38.1%), and Ambo referral hospital in Ethiopia (56%). In these studies, nurses consistently applied evidence-based pain management practices in post-operative care (16, 21–25). This variation may be attributed to differences in healthcare infrastructure, nurse-to-patient ratios, and access.

On the other hand, the level of post-operative pain practice in the current study was higher than the study conducted in Port Said city, Egypt in which only 10% of the nurses exhibit satisfactory practices (14) and the study conducted in Ghana in selected district hospitals in which only 2.4% of the nurses practiced standard pain management (26). This might be attributed to the differences in study period, hospital rank, and setting. Our study was conducted in higher-level public hospitals with better infrastructure and resources, including access to pain management protocols and essential medications. In addition, nurses in these settings are more likely to receive updated training and regular supervision, which together improve adherence to evidence-based practices.

In the current study, Female nurses were 2.5 times more likely to demonstrate good postoperative pain management practices compared to their male counterparts (AOR: 2.56; 95% CI: 2.1.78, 4.79; *P* < 0.001). While the underlying reasons for this difference are not fully understood, previous studies have also reported gender-related variations in nursing practice, suggesting that further research is needed to explore potential contributing factors. The finding is similar with the previous study conducted in Atlanta where female healthcare providers were found to demonstrate higher emotional intelligence and patient-centered approaches in pain management (27).

This study shows that nurses working in surgical units were about 3 times more likely to demonstrate effective pain management practices (AOR: 2.93; 95% CI: 1.27, 6.79; *P* < 0.0015). This finding might be due to the fact that surgical units expose nurses to post-operative patients who frequently require pain assessment and management, thus fostering expertise in this domain. This finding is similar with the study conducted in West Oromia, Ethiopia (28).

The current study showed that the odds of effective pain management were increased by 3 folds among nurses who used standardized pain assessment tools during pain management (AOR: 2.935; 95% CI: 1.65, 5.227; *P* < 0.0001). This might be due to the fact that pain assessment tools like the Numeric Rating Scale (NRS) provide quantifiable data that guide interventions and improve outcomes. This result is consistent with the previous study done in Vietnam (29).

In the current study, those nurses working in institutions with established pain treatment policies were 3.1 times more likely to practice effective post-operative pain management when compared with their counterparts (AOR: 3.1; 95% CI: 1.65, 5.86; *P* < 0.001). This shows the significance of organizational support in post-operative pain management. Policies ensure a systematic approach to pain management, reducing variability in practice. Studies in Colombia Ohio State align with this finding (30).

This study shows that nurses with training in post-operative pain management were over two times more likely to demonstrate standard post-operative pain management practices (AOR: 2.28; 95% CI: 1.46, 7.4). This finding underscores the significance of targeted training in equipping nurses with skills to address patient pain comprehensively. This finding aligns with the study done at University College Dublin, Ireland, which reported a significant improvement in pain management knowledge and practices following focused educational interventions (31).

## Conclusion

This study explored nurses' practices in postoperative pain management using direct observational checklists, which capture actual clinical behavior in real time and provide a more objective assessment compared to self-administered questionnaires. The findings revealed that only a quarter (25.72%) of observed nurses performed standard pain management. Factors positively associated with effective practice included female sex, use of standardized pain assessment tools, employment in hospitals with established pain management policies, work in surgical units, and prior training. To improve postoperative pain care, hospitals should expand regular, targeted training for nurses and implement standardized pain assessment tools across all units. all units, strengthen institutional policies, share best practices from surgical units with other departments, and address gender-related disparities through tailored professional development.

## Data Availability

The original contributions presented in the study are included in the article/Supplementary Material, further inquiries can be directed to the corresponding author.

## References

[1] Ceyhan D Güleç MS Ceyhan D Güleç MS. Postoperatif ağri sadece nosiseptif ağri midir? (is postoperative pain only a nociceptive pain?). Agri. (2010) 22(2):47–52. 10.1097/j.pain.0000000000001939 20582745

[2] Jungquist CR Vallerand AH Sicoutris C Kwon KN Polomano RC. Assessing and managing acute pain: a call to action. Am J Nurs. (2017) 117(3):S4–11. 10.1097/01.NAJ.0000513526.33816.0e 28212145

[3] Treede RD. The international association for the study of pain definition of pain: as valid in 2018 as in 1979, but in need of regularly updated footnotes. Pain Reports. (2018) 3(2):3–5. 10.1097/PR9.0000000000000643 PMC5902252 29756089

[4] Cox F. Basic principles of pain management: assessment and intervention. Nurs Stand. 25(1):36–9. 10.7748/ns2010.09.25.1.36.c7983 20949749

[5] Shanmugam R Assessment of postoperative pain management in mekelle public hospitals, Ethiopia. Int J Dev Res. (2018) 8(10):23843–23849.

[6] Smith J Roberts R Author B Roberts R. Pain fifth vital sign Vital, Wiley online Library (2015). Available online at: https://onlinelibrary.wiley.com/doi/10.1002/9781119139119.ch7 (Accessed January 12, 2026).

[7] Al-Shaer D Hill PD Anderson MA. Nurses’ knowledge and attitudes regarding pain assessment and intervention. Medsurg Nurs. (2011) 20(1):7–11. 21446289

[8] Chatchumni M Namvongprom A Eriksson H Mazaheri M. Thai nurses’ experiences of post-operative pain assessment and its’ influence on pain management decisions. BMC Nurs. (2016) 15(1):1–8. 10.1186/s12912-016-0136-8 26933384 PMC4772523

[9] World Health Organization (WHO). Statement on pain management guidance (2024). p. 1–2. Available online at: https://www.who.int/news/item/14-06-2019-statement-on-pain-management-guidance#:∼:text=WHO remains committed to working, prevent their misuse and harm (Accessed January 12, 2026).

[10] Cradock S Campbell I Tasker P Webb S Dick J. The role of the nurse in interdisciplinary pain management. Round Table Ser - R Soc Med. (2024) 60:27–34. Available online at: https://www.painnursing.it/the-role-of-the-nurse-in-interdisciplinary-chronic-pain-management/ (Accessed January 12, 2026).

[11] Bereka Negussie B Mulatu Gizachew E Belay Gizaw A Tegenu Lemma K Endale Mamo D. Post-operative pain assessment knowledge and practice among nurses working at Jimma University Medical Center, South West Ethiopia. Int J Africa Nurs Sci. (2022) 16(February):100406. 10.1016/j.ijans.2022.100406

[12] Dendir G Sintayehu A Anmut W. Knowledge, attitude and practice of nurses towards post-operative pain management in Wolaita Sodo University Teaching Referral Hospital, Ethiopia, institutional based cross-sectional study journal of anesthesia & clinical research. Anesth Clin Res. (2020) 11(January):1–9.

[13] Ninnoni JPK. Assessment and management of postoperative pain among nurses at a resource-constraint teaching hospital in Ghana (thesis/dissertation). (2019) 2019. 10.1155/2019/9091467 PMC6668527 31396418

[14] El sayed Mahedy N Mohamed MAE Mustafa FB. Nurses ‘ knowledge and practice regarding post-operative pain management for orthopedic patients. PSJN. (2021) 8(2):264–86.

[15] Adams SDM Varaei S Jalalinia F. Nurses' knowledge and attitude towards postoperative pain management in Ghana. Pain Res Manag. (2020) 2020:6765432. 10.1155/2020/4893707 PMC7429762 32831982

[16] Garimella V Cellini C. Postoperative pain control. Clin Colon Rectal Surg. (2013) 26(3):191–6. 10.1055/s-0033-1351138 24436674 PMC3747287

[17] Kizza IB Muliira JK. Nurses’ pain assessment practices and barriers to effective pain management among Ugandan surgical patients. J Pain Res. (2015) 8:479–486. 10.1186/s13104-017-2446-7

[18] Abebe T Abera M Tesfaye M. Nurses’ knowledge and attitudes towards pain management in Ethiopian hospitals. Ethiop J Health Sci. (2019) 29(5):577–584. 10.1186/s12912-024-02507-631666778

[19] Uman LS Stewart SH Chambers CT Cultural influences on the assessment and treatment of pain: a qualitative study. Pain Med. (2017) 18(10):1922–1931.

[20] World Health Organization. Ensuring Balance in National Policies on Controlled Substances: Guidance for Availability and Accessibility of Opioids for Pain Management. Geneva: WHO (2018).

[21] Thapa RD Gurung G. Nurses' knowledge, attitude and practice regarding postoperative pain management at selected hospitals, Bharatpur, Nepal. J Chitwan Med Coll. (2020) 10(1):64–8.

[22] Umuhoza O Chironda G Katende G Mukeshimana M. Perceived knowledge and practices of nurses regarding immediate post - operative pain management in surgical wards in Rwanda. A descriptive cross - sectional study. (2019) 10(1):145–51. Available online at: https://www.sciencedirect.com/science/article/pii/S2214139118300635 (Accessed January 12, 2026).

[23] Teshome ZB Aychew Y Mitiku W Guta B. Level of attitude, knowledge and practice of nurses toward postoperative pain management, cross-sectional study. Ann Med Surg. (2022) 84(November):104902. 10.1016/j.amsu.2022.104902 PMC9758382 36536707

[24] Feleke DG Chanie ES Tassaw SF Dires T. Practice of non-pharmacological post-operative pain management and associated factors among nurses working in public referral hospitals of Amhara regional state, Ethiopia, 2019. Int J Africa Nurs Sci. (2024) 20(3):100693. 10.1016/j.ijans.2023.100642

[25] Dechasa A Kurke A Abdisa D Gurmu Y. Post-operative pain management practice and associated factors among nurses working at public hospitals, in Oromia region, Ethiopia. *medRxiv* [Preprint]. *2022.04.14.22273889* (2022). Available online at: http://medrxiv.org/content/early/2022/04/17/2022.04.14.22273889.abstract (Accessed January 12, 2026).

[26] Menlah A Garti I Amoo SA Atakro CA Amponsah C. Knowledge, attitudes, and practices of postoperative pain management by nurses in selected district hospitals in Ghana. (2018) 4:1–11. 10.1177/2377960818790383 PMC7774443 33415201

[27] Strekalova YA Kong S Kleinheksel AJ Gerstenfeld A. Gender differences in the expression and cognition of empathy among nursing students: an educational assessment study. Nurse Educ Today. (2019) 81(October 2019):1–6. 10.1016/j.nedt.2019.04.004 31295661

[28] Mekonen WM Muhye AB Gobeza MB. Nurses’ knowledge and practice about neonatal pain management in public hospitals in West Oromia, Ethiopia, 2022: multi-centered cross-sectional study. BMC Nurs. (2024) 23(1):1–11. 10.1186/s12912-024-01972-3 38724983 PMC11080202

[29] Gárriz ÁS Mula JM Montoya P Riquelme I. Pain assessment tools in adults with communication disorders : systematic review and meta - analysis. BMC Neurol. (2024) 24(1):66. 10.1186/s12883-024-03539-w 38368314 PMC10873938

[30] Micha R. 乳鼠心肌提取 HHS public access. Physiol Behav. (2017) 176(1):100–106. Available online at: https://pmc.ncbi.nlm.nih.gov/articles/PMC8098053/pdf/nihms-1694132.pdf (Accessed January 12, 2026).

[31] Reaza-Alarcón A Rodríguez-Martín B. Effectiveness of nursing educational interventions in managing post-surgical pain. Systematic review. Investig y Educ en Enferm. (2019) 37(2):e10. Available online at: https://pmc.ncbi.nlm.nih.gov/articles/PMC7871485/pdf/2216-0280-iee-37-02-e10.pdf (Accessed January 12, 2026). 10.17533/udea.iee.v37n2e10 PMC7871485 31487447

