# Supplementary material for: Exploring post-operative pain management practices for improved outcomes among nurses in public hospitals in West Shewa, Ethiopia: a multicenter observational study
Source: Front Pain Res (Lausanne). 2026 Jan 30;6:1571968. doi: 10.3389/fpain.2025.1571968 (PMC12901455; doi:10.3389/fpain.2025.1571968)
Supplement: Supplementary file 1 [file Datasheet1.pdf]

Observational checklist to assess nurses' POP management practices

Ward; ________ Participant code.______________ Bed no.___, __, ___,___,___,___.

Direction: tick in the space provided under "Yes" when the nurse performed the POP management practice in front of each item and tick under "No" for not practiced item during the observations.

S. n	Structured Observational check list format	Frequency of observation						
		One		Two		Three		Total
	Variables	yes	No	yes	No	yes	No	
1	Does he/she assess pain by means of pain scales for patients able to communicate?							
2	If yes, what type of pain scale used<br>☐Verbal rating scale(VRS) ☐Numerical rating scale(NRS)<br>☐Visual analogue scale(VAS) ☐Faces pain scale(FPS)<br>☐Behavioral methods ☐Physiological methods							
3	Does he/she document the result of pain assessment?							
4	Does he/she provide ordered medications around the clock?							
5	Provide pain medication prior to painful events (such as dressing or wound healing/caring)?							
6	Does he/she involve patients in pain management process?							
7	Evaluate the effect of a given treatment by reassessing pain before and after treatment intervention?							
8	Provide clean, calm, and a well-ventilated ward environment for pain management?							
9	Lay patients on neat, well-laid bed postoperatively?							
10	Encourage early ambulation/exercise with analgesia?							
11	Quieting and consoling the patients to reduce pain?							
12	Does he/she use non pharmacological methods of POP relief?							
13	If used what type of method?							

	☐Massage and stretching<br>☐Positioning:<br>☐Emotional Support(e.g. presence or being with)<br>☐Comforting/Reassurance<br>☐Supportive touch to reduce pain.<br>☐Thermal Regulation (Heat and Cold application)<br>☐Use patient distraction and Relaxation to reduce POP.<br>☐Other(specify)__________							
	Total score							
