# Supplementary material for: Exploring post-operative pain management practices for improved outcomes among nurses in public hospitals in West Shewa, Ethiopia: a multicenter observational study
Source: Front Pain Res (Lausanne). 2026 Jan 30;6:1571968. doi: 10.3389/fpain.2025.1571968 (PMC12901455; doi:10.3389/fpain.2025.1571968)
Supplement: Supplementary file 2 [file Datasheet2.pdf]

Appendix II: Questionnaire

Part I: Socio Demographic Characteristics of respondents

Instruction: Please circle the number of your choice.

It. no.	Questions	Responses	Remark
101	Sex	1. Male 2. Female	
102	How old are you?	------------years	
103	What is your ethnicity	1. Amhara 2. Oromo<br>3.Tigre 4.Others________	
104	What is your marital status?	1. Married 2. Single<br>3. Divorced 4. Widowed	
105	What is your religion?	1. Orthodox 2. Muslim<br>3. Protestant 4. Catholic<br>5.Others(specify)___________	
106	What is your level of Qualification?	1. Diploma 2. Bachelor degree<br>3. Master's degree and above	
107	How many years of work Experience do you have?	1. ________________	
108	How long had you been working in postoperative area	1._______________	
109	Where is your current area of Practice?	1.________________	
110	Have you received any training related to POP management?	1. Yes<br>2. No	If No Skip to Q12
111	If yes, How do you receive training?	1. Lecture 2. Course<br>3. Conference 4. Workshop	
112	Do you have access to read pain management guidelines in your hospital?	1. Yes<br>2. No	If No Skip to

			next
113	If yes, How often you read guidelines?	1 Always 2 Monthly<br>3 Quarterly 4 Yearly	

Part II: Respondents knowledge to POP assessment and management related questions

Instruction: Please circle the number of your choice.

Item No.	Items	Response
201	When a patient requests increasing amounts of analgesics to control pain, this usually indicates that the patient is psychologically dependent.	1. Yes<br>2. No<br>3.not sure
202	Vital signs are always reliable indicators of the intensity of a patient's pain.	1. Yes<br>2. No<br>3.not sure
203	Pain assessment includes onset, duration, variability, location, and intensity of pain.	1. Yes<br>2. No<br>3.not sure
204	When using the WHO pain ladder to treat acute pain, treatment should go from bottom to top.	1. Yes<br>2. No<br>3.not sure
205	combining analgesics that work by different mechanisms may result in better pain control with fewer side effects than using a single analgesic agent	1. Yes<br>2. No<br>3.not sure
206	Pain should be assessed before and after administering pain drugs.	1. Yes<br>2. No<br>3.not sure
207	observation is part of the method used in surgical pain assessment	1. Yes<br>2. No<br>3.not sure

208	The side effects of narcotics should be observed at least 20 minutes after Administration	1. Yes<br>2. No<br>3.not sure
209	The recommended route of administration of opioid analgesics with brief, severe pain of sudden onset such as POP is intramuscular.	1. Yes<br>2. No<br>3.not sure
210	Analgesics for POP should initially be given around the clock on a fixed schedule.	1. Yes<br>2. No<br>3.not sure
211	Pre surgery injection such as anesthesia is given for pain management	1. Yes<br>2. No<br>3.not sure
212	Respiratory depression rarely occurs in patients who have been receiving stable doses of Opioids over a period of months.	1. Yes<br>2. No<br>3.not sure
2013	Opioids should not be used in patients with a history of substance abuse.	1. Yes<br>2. No<br>3.not sure
214	Rating scale ranging from (0) “no pain at all to (10) the worst pain” is essential to adopt in pain assessment.	1. Yes<br>2. No<br>3.not sure
215	If a patient sleeps with no movement postoperatively, this indicates that patient is not in pain.	1. Yes<br>2. No<br>3.not sure

Part III: Respondents attitude to POP assessment and management related questions Instruction:

Please click the box you choose

Item no	Questions	Response		
		Agree	Don't know	Dis agree
301	Your patient should experience discomfort prior to giving the next dose of pain medications.			
302	Distraction can reduces pain intensity			
303	A patient's spiritual beliefs may lead them to think pain and suffering are necessary.			
304	Using pain measurement instruments is integral in postoperative pain management.			
305	Morphine is very strong drug; patients in postoperative pain would be content with just one dose.			
306	Nurses are best judges of the patient's pain intensity because they spent 24 hours with the patients			
307	Lack of pain expression does not mean lack of pain.			
308	Effective analgesia is an essential part of postoperative Management			
109	Pain is what the patient says it is.			

Part IV: Items to assess practice

Direction: Read the following questions carefully and encircle on your choice. If your choice is "yes" tick also on how frequently you practice it and skip to the next questions if your choice is "never".

401	Do you assess pain for patients able to communicate?<br>If your choice is never skip to Q4	1.yes<br>2.never	If yes how frequently?<br>☐Always ☐Sometimes
402	Do you use a pain assessment tool for pain scale?<br>If never used go to Q404	1.yes<br>2.never	If yes how frequently?<br>☐Always ☐Sometimes
403	If use, Please! Name the tool(s) you used.	______________, _______________	
404	If your answer for Q401 & 402 above is never, What Was the barriers that hinders you from pain assessment? You can choose multiple options.		1. Nursing workload<br>2.Lack of standard pain assessment tool in hospital<br>3.lack of training on pain management<br>4. Lack of pain management Guideline in the hospital<br>5.Patient inability to communicate<br>6.Other(specify)________
405	Do you encourage use of transcutaneous electrical nerve stimulator for pain management	1.yes<br>2.never	If yes how frequently?<br>☐Always ☐Sometimes
406	Do you combine opioids with NSAID's rather than single analgesic agents when managing POP as suggested by World Health Organization?	1.yes<br>2.never	If yes how frequently?<br>☐Always ☐Sometimes
407	Do you document the findings after pain assessment?	1.yes	If yes how frequently?

		2.never	☐Always ☐Sometimes
408	If your answer for Q407 is never, what was the reason for your not documenting the finding? If not choose never, go to Q409.<br>You can choose multiple options		1. Nursing workload<br>2. No designed area for charting<br>3.Lack of familiarity with the assessment tools<br>8.Other(specify)________
409	Do you encourage prayer by patients or religious leader postoperatively?	1.yes<br>2.never	If yes how frequently?<br>☐Always ☐Sometimes
410	Do you administer ordered pain medication, around the clock (regularly) as ordered?	1.yes<br>2.never	If yes how frequently?<br>☐Always ☐Sometimes
411	Do you use music therapy to reduce postoperative pain?	1.yes<br>2.never	If yes how frequently?<br>☐Always ☐Sometimes
412	Do you reassess pain after giving pain medication to evaluate the effectiveness of pain medication?	1.yes<br>2.never	If yes how frequently?<br>☐Always ☐Sometimes
413	After surgery, do you provide comfortable positions to help relieve pain?	1.yes<br>2.never	If yes how frequently?<br>☐Always ☐Sometimes
414	Do you ask and help to support the painful areas when the patients moving or coughing after surgery?	1.yes<br>2.never	If yes how frequently?<br>☐Always ☐Sometimes
415	Do you provide clean, calm, and a well-ventilated ward environment for POP management?	1.yes<br>2.never	If yes how frequently?<br>☐Always ☐Sometimes
416	Do you lay the patients on neat, well-laid bed postoperatively?	1.yes<br>2.never	If yes how frequently?<br>☐Always ☐Sometimes
417	Do you encourage massaging and stretching to reduce POP?	1.yes<br>2.never	If yes how frequently?<br>☐Always ☐Sometimes
418	Do you apply heat and cold compresses to manage postoperative pain?	1.yes<br>2.never	If yes how frequently?<br>☐Always ☐Sometimes
419	Do you encourage early ambulation/exercise with analgesia?	1.yes<br>2.never	If yes how frequently?<br>☐Always ☐Sometimes
420	Do you encourage use of acupuncture?	1.yes	If yes how frequently?

		2.never	☐Always ☐Sometimes
421	Do you use patient distraction, relaxation, and guided imagery postoperatively to reduce pain?	1.yes<br>2.never	If yes how frequently?<br>☐Always ☐Sometimes
422	Do you dress, bandage, splint, and reinforce wound sites postoperatively?	1.yes<br>2.never	If yes how frequently?<br>☐Always ☐Sometimes
